# Bacteriophages as Food Biocontrol Agents: A One Health Framework for Manufacturing Quality, Regulatory Governance, and Ethical Stewardship—A Narrative Review

**DOI:** 10.3390/v18030368

**Published:** 2026-03-16

**Authors:** Rafail Fokas, Panos G. Kalatzis, Apostolos Vantarakis

**Affiliations:** 1 Department of Public Health, Medical School, University of Patras, 26504 Patras, Greece; 2 Center for Evolutionary Hologenomics, Globe Institute, University of Copenhagen, Øster Farimagsgade 5, 1353 Copenhagen, Denmark; panos.kalatzis@sund.ku.dk

**Keywords:** bacteriophages, One Health, food safety, regulatory, ethical governance, antimicrobial resistance

## Abstract

Introduction: Bacteriophages are emerging as viable food safety tools, yet their global implementation is hindered by regulatory fragmentation and a lack of harmonized data standards. This review addresses the gap between scientific maturity and governance readiness by evaluating manufacturing quality, safety requirements, and international oversight frameworks. Methods: A narrative review was conducted through a structured search of databases including PubMed, Scopus, Web of Science, and Google Scholar (up to December 2025). We analyzed scientific research and publicly available regulatory documents from agencies such as the FDA, EFSA, USDA, Health Canada, and FSANZ to identify authorization routes and manufacturing standards. Results: Commercial phage products are primarily approved as processing aids in jurisdictions like the United States, Canada, and Australia/New Zealand. We identified convergent technical requirements across these regions, including genomic integrity (absence of toxins and antimicrobial resistance genes), purity, potency, and matrix-validated efficacy. However, significant gaps remain in unified terminology, environmental risk assessment, and post-market monitoring for resistance emergence. Conclusions: To facilitate global adoption, a One Health-oriented governance cycle is proposed. This includes establishing interoperable phage seed banks, standardized dossier formats, and adaptive lifecycle controls (phagovigilance) to ensure long-term efficacy and public trust.

## 1. Introduction

Antimicrobial resistance (AMR) continues to reduce antibiotic efficacy in clinical, agricultural, and environmental settings, exposing structural flaws in current hazard control tactics and boosting demand for biologically based alternatives [1]. In this context, bacteriophages (phages)—viruses that infect and lyse bacteria with excellent host specificity—have resurfaced as precision biocontrol techniques capable of lowering pathogen burdens in food systems while minimizing disruption of commensal microbiota and avoiding chemical residues [2,3]. These phage-based treatments are attractive complements to, rather than replacements for, established sanitation and antimicrobial programs due to their ubiquity in food-relevant environments [4], ability to replicate self-limitingly in the presence of susceptible hosts [5], and compatibility with cold-chain and surface applications [6].

However, the move from laboratory proof-of-concept to everyday, permitted use is unequal. Specifically, despite increasing scientific and commercial momentum, scientific maturity has exceeded governance readiness. Several jurisdictions have approved specific phage preparations for specific food applications, most commonly as food processing aids [7], although others route identical items through neighboring categories such as feed additives or microbial biocontrol agents, or they lack specified channels entirely [2,8]. Furthermore, expectations for genomic characterization (including the elimination of lysogeny modules, toxins, and antimicrobial resistance genes), manufacturing quality and batch consistency, environmental risk assessment, and labeling claims vary significantly between authorities [9]. As a result, comparable items are assessed using non-equivalent criteria, complicating mutual recognition and inhibiting investment in scalable production, quality analytics, and post-market monitoring infrastructure.

This governance gap is inherently One Health-relevant, because the benefits and uncertainties of live biocontrol agents extend across human, animal, and environmental interfaces [10]. When applied to phage-based food biocontrol, the One Health paradigm expands the focus beyond antimicrobial efficacy to include environmental persistence, selection forces that may promote resistance, occupational and consumer exposures, and the transparency duties of food business operators and regulators [11]. Because evidence on host range, genome content, stability, and real-world performance evolves over time, live biocontrol agent governance must be adaptive. Accordingly, pre-market evaluations should focus on clearly defined risk-relevant endpoints, while post-market monitoring should be capable of detecting efficacy loss, resistance emergence, and unforeseen ecological effects, thereby triggering proportionate mitigation or reformulation [12,13,14]. Ethical and cultural issues, such as stewardship obligations, acceptable labeling methods, and credible corrective action processes, are critical to preserving public trust in adaptive supervision.

This review begins with the notion that phage-based treatments in food systems may only reach industrial scale if scientific practice, manufacturing quality, regulatory design, and ethical governance are all aligned within a One Health-oriented cycle of learning and stewardship. We begin by summarizing essential biological properties of phages pertinent to food applications, as well as their present commercial utilization across the farm-to-fork continuum. We then look at manufacturing quality and product characterization criteria, pre-market risk assessment using a One Health approach, and the global regulatory landscape for food-related phage products. Based on this analysis, we identify major challenges and limitations, discuss ethical and societal governance issues, and finally propose a practical roadmap for international harmonization of data standards and oversight approaches, allowing bacteriophage technologies to contribute transparently and at scale to food safety and AMR mitigation.

## 2. Materials and Methods

A structured literature and regulatory source search was performed to identify scientific and regulatory information on bacteriophages as food biocontrol agents, with a focus on manufacturing quality, One Health implications, and authorization routes. To ensure comprehensive coverage, PubMed, Scopus, Web of Science, and Google Scholar were searched from inception to 13 December 2025. The search strategy utilized combinations of terms such as “bacteriophage”, “phage biocontrol”, “food safety”, “processing aid”, “regulation/approval/authorization”, “One Health”, and “environmental fate”, which were combined with AND/OR adapted to each database syntax. In parallel, publicly available regulatory documents and official databases/websites were screened for positions, classifications, and approvals relevant to food-related phage applications. This included evidence from the European Medicines Agency (EMA), European Food Safety Authority (EFSA), the European Commission (EU food laws/authorizations where applicable), Food and Drug Administration (FDA), United States Department of Agriculture (USDA), US Environmental Protection Agency (EPA) (biopesticide/biocontrol route where applicable), Health Canada and Food Standards Australia New Zealand (FSANZ), as well as international guidance sources used for harmonization and quality. Records were considered eligible for inclusion if they addressed phage use in food matrices or food contact contexts, as well as regulatory review, health/environmental factors, standardization, exposure assessment, or post-market oversight. Titles/abstracts were examined, followed by full-text analysis, and evidence was thematically integrated into the review’s sections.

## 3. Bacteriophages in Food Systems: Mechanisms and Biology

The narrative synthesis presented in the following sections (3 through 8) represents the findings derived from the structured search strategy detailed in Section 2. To maintain a focused analysis on biocontrol stewardship, sources were included based on their relevance to matrix-validated efficacy, manufacturing standards, and regulatory frameworks, while excluding studies without direct food safety application.

**Figure 1:**
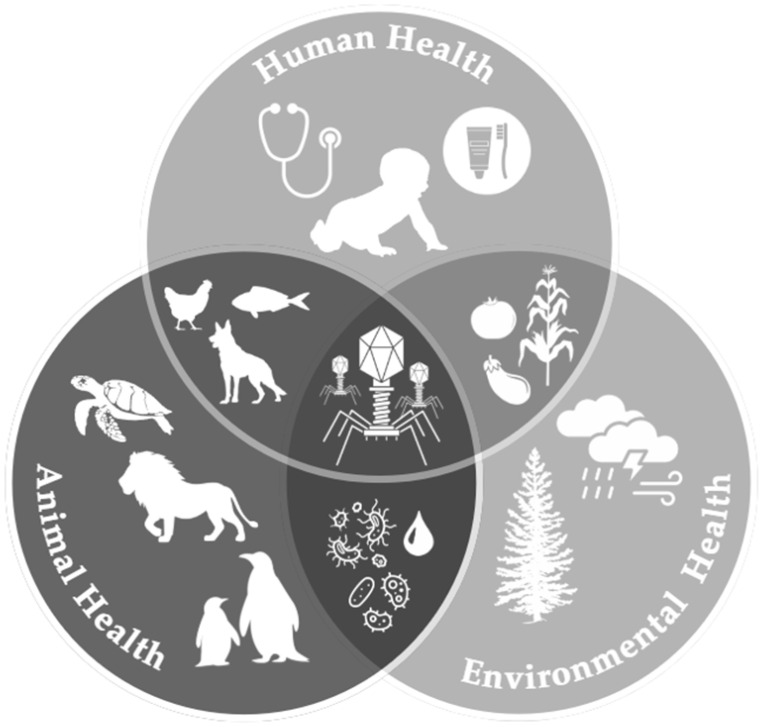
One Health domains relevant to bacteriophage biocontrol.

Phages are viruses that infect bacteria with high specificity, making them effective precision weapons for targeting foodborne pathogens without hurting beneficial microbiota [15]. Phages, unlike broad-spectrum chemical antimicrobials, leave no chemical residues and do not affect food’s organoleptic qualities [16]. This aligns with One Health principles of ecological sustainability and consumer safety. As shown in Figure 1, these domains are intrinsically linked in the context of biocontrol.

Regarding their biology, phages may follow the lytic or lysogenic lifecycle. In the former, a virulent (lytic) phage adsorbs to a vulnerable bacterial host, injects its genome, and hijacks the host’s machinery to make offspring virions, resulting in bacterium lysis and the release of new phages. In the latter, the genome of the temperate phages may be integrated in the bacterial chromosome until an external stressor triggers its induction and therefore activation of the lytic pathway. Biocontrol requires exclusively lytic (virulent) phages, as the integration of temperate phages into host genomes may contain unwanted genes [17]. In practice, potential phages for food applications are subjected to rigorous genomic and phenotypic characterization: they must lack integrase, recombinase, or lysogeny genes, as well as be free of recognized toxin, virulence, or antibiotic resistance determinants [18,19]. This ensures that the phage does not bring detrimental features into the bacterial population. Furthermore, phages are evaluated for broad activity against epidemiologically important strains of the target pathogen [20]. Using in vitro isolates from various food and processing contexts, host range experiments can find phage cocktails that cover a diverse range of pathogen variations. Individual phages have a limited host range, which can be both advantageous (precise targeting) and disadvantageous (infecting only specific strains). The environment also significantly influences phage biology in food systems. Phage adsorption and replication kinetics can be influenced by several matrix variables such as temperature, pH, and water activity [3]. Phages are most active at specific temperature ranges. When applied to chilled foods, low temperatures may inhibit their activity, while excessive heat (e.g., cooking) might inactivate them [21]. As a result, successful biocontrol necessitates the selection or formulation of phages that remain stable and active under the target food’s relevant parameters (for example, pH of produce washes, salinity of brines, refrigeration temperatures). Stability tests typically assess phage infectivity following exposure to processing-related stressors such as mild heat, acid, or desiccation. To mitigate these effects, protective formulation methods, such as adding buffering agents, salts, or sugars to phage formulations, can improve stability during storage and application [22]. Indeed, studies have shown that adding stabilizers such as trehalose or certain polymers can keep phages alive during drying or temperature changes [23]. In terms of delivery, phages can be supplied to foods in a variety of ways, including liquids (sprays, dips, or mists) or incorporated in gels and coatings if moisture addition is minimized [24]. The delivery technique was designed to increase phage interaction with the target bacterium. For example, an aqueous spray may be perfect for surfaces, guaranteeing equal coverage, whereas phage mixed into rinse water can constantly treat items such as poultry carcasses during processing.

Crucially, phages require metabolically active bacterial hosts to reproduce [25]. This means timing and integration into the food manufacturing process are important. Phages are frequently most effective when given at a time when the target bacteria are in the development phase (for example, on freshly contaminated surfaces prior to a cool stage). If administered too late (for example, after infections have been fully inactivated by another barrier or are starving), phage multiplication may be limited, confining their impact to a single binding and lysis event [26].

Phages’ self-amplification is a two-edged sword. In a favorable environment with susceptible bacteria, phages can grow and maintain their efficiency, sometimes requiring only a little initial dose to accomplish large bacterial reduction. This self-propagating property is useful for residual action (for example, a phage left on a food surface may continue to lyse newly emerging bacteria during storage). In well-managed food production, phage replication may be limited, requiring a high dose to accomplish the intended death. Applying a high phage titer is crucial for quick efficacy at low contamination levels [27]. The notion of effective multiplicity of infection (MOI) in foods recognizes that due to complex food surfaces and micro-environments, only a percentage of applied phages will successfully interact and infect bacteria. Empirical experiments on various foods (e.g., meats, vegetables, dairy) are used to identify the ideal phage dose to reliably achieve a pathogen decrease.

Another critical constraint is that bacteria can evolve resistance to phages [28]. Bacteria can resist phage attack by modifying surface receptors, creating inhibitors of phage DNA injection, or using CRISPR-Cas systems [29]. While resistance may sometimes be phage-specific, bacteria can also harbor broader innate and adaptive defense systems. Therefore, phage cocktails targeting distinct receptors can reduce the probability of rapid escape, but they do not eliminate resistance risk and require verification under intended use conditions. This reasoning supports the use of multi-phage products: if a bacterium mutates, it may still be receptive to the other phage(s) in the cocktail. Furthermore, phage resistance can reduce bacterial fitness or pathogenicity, and in food settings, resistant strains may be less competitive or detectable using monitoring methods [30]. Here, “persistent strains” refers to recurrent isolates of the target pathogen recovered through routine environmental monitoring in processing environments (e.g., from food contact surfaces or equipment). Regular susceptibility testing is intended to verify that the deployed phage preparation remains active against the target contaminant under the intended conditions of use. Evidence of reduced susceptibility would trigger predefined corrective actions (e.g., intensified sanitation and/or cocktail update), whereas persistence despite susceptibility may indicate operational factors (e.g., insufficient contact time, organic load, or application parameters) rather than loss of phage activity. Where environmental release beyond the facility is plausible (e.g., via wastewater), monitoring and risk management should be proportionate to the exposure scenario.

Phages are typically considered safe to consume and handle [31]. Phages are naturally found in the environment and foods, and humans consume billions of them on a daily basis with no known negative consequences. Safety and toxicological evaluations performed for certain food-use lytic phage preparations have not identified acute adverse effects under the tested conditions [32]; however, phage–host–microbiome interactions can be context-dependent, and broad claims of ‘no negative effects’ are not appropriate across all settings [33]. Rodents and primates given high doses of *Listeria*-targeting phages (e.g., ListShield™ and P100) had no significant clinical or histopathological consequences [34]. A study on human volunteers found no significant changes in gut microbiota or health effects after consuming an *E. coli*-specific T4 phage [35]. These findings lend support to the idea that lytic phages have an essentially safe profile. However, because phages are self-replicating biological agents, safety studies go beyond standard chemical toxicology. Important issues include ensuring that the phage’s genome does not encode any damaging proteins (thus the genomic analyses outlined above) and that the phage does not unintentionally enable horizontal gene transfer across bacteria. Although strictly lytic phages do not typically facilitate transduction, some can occasionally package and transport pieces of host DNA to other bacteria (generalized transduction) [36]. Risk assessments analyze the possibility of phage application spreading genes such as antibiotic resistance genes (ARGs) amongst bacteria, but this is uncommon [37]. To reduce this danger, phage strains should be carefully selected (avoiding those known to transduce) and tested under simulated use settings (for example, combining phages with high densities of bacteria to look for gene transfer events).

## 4. The State of the Art: Commercial Applications and Farm-to-Fork Integration

Over the last two decades, numerous phage-based products targeting common foodborne pathogens have entered the market, indicating that phage technology has progressed from laboratory research to real-world applications [38]. The United States is at the forefront of these advancements. ListShield™, a cocktail of six lytic phages against *Listeria monocytogenes*, was the first FDA-cleared phage preparation for food safety [39]. It was designated as Generally Recognized as Safe (GRAS Notice No. 528) for ready-to-eat foods in 2006 [40]. Additional products, such as EcoShield™ (targeting *E. coli* O157:H7 on red meat) [41] and SalmoFresh™ (targeting *Salmonella* spp. on poultry and produce), were developed as a result [42].

Listex™ P100, a phage against *Listeria*, was developed in the Netherlands and approved as a processing aid by FSANZ in 2011 [43]. Listex P100, marketed as PhageGuard Listex, has received favorable evaluation from EFSA for certain purposes [44]. Micreos Food Safety (NL) has released PhageGuard-E and PhageGuard-S, two phage products that target *E. coli* O157 and *Salmonella*, respectively. In 2018, the FDA and USDA certified PhageGuard-E as GRAS for use on cattle, permitting its application as a “smart, green, and easy to apply solution” for surface disinfection of beef carcasses and trimming [45]. These licenses demonstrate North America’s regulatory acceptance of phages as processing aids—chemicals employed during processing but not found in large quantities in the final product. To provide a more comprehensive overview, Table 1 lists numerous noteworthy commercial phage products, their targets, and authorized applications in various countries (including GRAS notifications in the United States and comparable clearances elsewhere). The products listed are intended as a representative, regulatory-anchored snapshot based on publicly documented authorizations/notifications, rather than an exhaustive inventory of all pilot and field applications reported in the literature.

These items show that phage biocontrol has progressed beyond theory, since they are being used at numerous stages along the food supply chain. Most are designed as liquids (sprays or dips) with high concentrations of phages (usually 10^7^-10^9^ PFU/mL) and are put directly onto foods or into food contact settings. The target pathogens, *Listeria*, *Salmonella*, *E. coli* O157, and *Shigella*, are among the most common causes of foodborne illness and recalls. *Listeria monocytogenes*, which may thrive at refrigerator temperatures on ready-to-eat (RTE) foods, has been a key focus [59]. Phage treatments such as ListShield™ and Listex™ P100 can effectively suppress *Listeria* on deli meats, smoked fish, and soft cheeses. These phages have been demonstrated in trials to considerably decrease *Listeria* on treated surfaces (typically attaining 1–2 log CFU/cm^2^, with some studies showing up to 5 log reductions under optimum circumstances) [59,60,61].

Phages are utilized in poultry processing, such as SalmoFresh on raw chicken parts to lower *Salmonella* levels by ~1–2 log CFU on skin surfaces, boosting current sanitation methods [62]. In beef grinding processes, phages targeting *E. coli* O157 are used to prevent this disease from entering ground beef [63].

Beyond these specific product applications, there is rising interest in strategically using phages throughout the food production continuum—from farm to processing plant to retail—as part of a comprehensive food safety system. Phages are being investigated at the farm level as replacements or adjuncts to antibiotics in agriculture. For example, the BAFASAL^®^ phage cocktail (mentioned above) is provided to poultry (by drinking water or spray) to minimize *Salmonella* colonization in live chickens [64].

Phage supplementation has been shown in field studies on commercial broiler farms to reduce *Salmonella* levels in chicken litter and carcasses after slaughter [65]. By reducing pathogen load on the farm, the danger of downstream contamination in the slaughterhouse is lowered, this pre-harvest biocontrol strategy is consistent with the One Health concept of acting at many stages [66]. Another example is aquaculture, where phages have been used to manage *Aeromonas*, *Flavobacterium* and *Vibrio* diseases in fish and shrimp farms, increasing animal health and minimizing the transmission of these germs to process water. While not yet commercialized, such applications are actively being researched [67,68].

Phages are increasingly being used as preventative or corrective measures in HACCP plans in processing industries [69]. A strategy is to use phages in sites where contamination is expected or has been found. For example, in a fresh fruit packaging line, if *Listeria* is found in a wash water sample (environmental monitoring), an appropriate phage might be injected to the wash system to swiftly lower the bacterial load, supplementing physical sanitizers. During pauses in meat production, phages may be sprayed on conveyor belts or slicers to target niches where bacteria create biofilms that withstand normal cleaning. Phages have a unique capacity to penetrate biofilms (often more effectively than chemical sanitizers) and can be used with biofilm-disrupting agents (such as enzymes/essential oils) to provide a synergistic effect [34,70,71,72]. Phage interventions can be integrated across the food chain at four critical control points: (1) on the farm (prophylactic phage in animal feed or spray) [73], (2) at slaughter (spraying carcasses or processing equipment) [74], (3) during post-process handling (treating food contact surfaces like cutting tables or directly applying to foods like produce or cheese) [75] and (4) at packaging [76].

When implementing these approaches, practical questions emerge. Where and when should phages be used for maximum effect? How can their impact be verified? Phage performance in food and processing environments is strongly influenced by application context, including temperature, organic load, surface moisture, contact time, and the physiological state of the target bacteria. For example, refrigerated temperatures can limit in situ efficacy on meat matrices compared with warm in vitro conditions; therefore, matrix- and context-validated protocols are necessary under intended use conditions [77]. Accordingly, timing relative to process steps is key: one successful model is to treat RTE foods post lethality, just before packaging, to kill any contaminants that escaped cooking or entered during handling. This is exactly how phages are employed in some deli meat facilities: after cooking and chilling but before the product is packaged, a mist of *Listeria* phages is sprayed [44,78]. Verification should be integrated into routine food safety verification activities, including product testing across shelf life and environmental monitoring. A consistent reduction in prevalence and/or levels of the target pathogen in treated batches relative to appropriate controls supports effectiveness. In addition, environmental monitoring can be used to assess performance at recurrent contamination sites (e.g., drains or equipment-associated niches) by tracking target pathogen detection trends before and after implementation, alongside sanitation and process controls.

Beyond processing, phage applications in distribution/retail are more limited but may remain relevant for certain high-risk refrigerated foods (e.g., Listeria-targeting preparations on soft cheese or smoked fish), where labeling/disclosure considerations can become more salient and jurisdiction-dependent [79,80].

In many countries, phages that are categorized as processing aids and utilized in small doses with no functional impact during ingestion do not need to be labeled. However, if labeling is needed or desired (e.g., for marketing openness), it must be properly written, often stating the phage preparation as an ingredient (e.g., “contains bacteriophage preparation (phages targeting *Listeria*).”). It should not imply that phages are used to treat diseases. Consumer perception studies suggest that when consumers learn about phages and why they are utilized, their acceptability of phage-treated food increases. In fact, one survey found that consumers were, on average, willing to pay more for phage-treated produce if it meant improved safety, especially once they understood that phages are natural and fight bacteria [81].

Several case studies demonstrate cutting-edge integration. In 2019, a big poultry processor applied SalmoFREE^®^, a commercial phage cocktail, via spraying in a broiler house and found a drop in *Salmonella* prevalence in chickens entering the processing facility [82]. This resulted in decreased *Salmonella* contamination in processed items. Another example is in product packing: a fresh-cut produce firm added a phage rinse (a cocktail against *E. coli* O157) for leafy greens. According to published findings, this resulted in large decreases in pathogen numbers on inoculation leaves without impacting crop sensory quality [83]. Furthermore, in facilities with ongoing *Listeria* issues (e.g., a smoked fish plant), routine phage applications in environmental hotspots (floors, drains, cold rooms) have been shown to reduce the frequency of *Listeria* positives over time, acting as an additional barrier alongside sanitation [84]. These findings demonstrate that, when appropriately incorporated, phages can help to significantly reduce risk.

## 5. Manufacturing Quality and Product Characterization

Bacteriophage manufacture involves large-scale co-culture of the phage with its bacterial host, similar to biopharmaceutical fermentation. For food biocontrol, commercial phage preparations are typically formulated as liquid concentrates (buffers) intended for spray/dip/rinse application to foods or food contact surfaces [78], and increasingly as stabilized formats (e.g., refrigerated liquids or dry/encapsulated preparations) to support shelf life and operational handling [85]. Phages are cultivated in liquid culture under controlled circumstances, harvested, concentrated (by centrifugation and filtration), and purified to eliminate bacterial debris, leftover nutrients, and contaminants. Phage isolates are often manufactured individually and formulated as single-phage medicines or blended in defined cocktails to broaden host coverage. Quality control (QC) throughout the production process is critical to ensuring reproducible product identity and consistency. The EMA draft quality guidelines for phage therapeutic products recommend a two-tiered seed bank approach, with a Genetically Verified Master Seed Lot and Working Seed Lots derived from a single clonal phage strain. Advanced approaches, such as complete genome sequencing to establish identity and discover genetic variation, and orthogonal testing to verify plaque morphology and host specificity, are required to thoroughly characterize each seed lot and subsequent production batch. Manufacturers should ensure the phage’s genetic stability during scale-up by sequencing numerous batches against the master seed sequence to prevent undesired alterations during propagation. In practice, this may require routine whole-genome sequencing or high-resolution genetic fingerprinting, along with phenotypic testing (e.g., plaque shape and clarity), to verify the viral particle and its lytic activity stay constant over time [86].

Identity testing for phage products usually combines molecular and phenotypic approaches. The EMA guideline suggests utilizing highly specific assays (e.g., qPCR or WGS) to differentiate therapeutic phages from those produced in the same facility. The phage genome is mapped using next-generation sequencing to identify open reading frames and give taxonomic categorization [87]. This analysis screens for unwanted genetic elements, such as genes for lysogeny, toxins, prophages, or antibiotic resistance determinants, which must be absent or justified by a detailed risk assessment. EMA recommendations specify that any phage genomic sequence encoding antibiotic resistance or virulence factors is a harmful contaminant and should be avoided until rigorously inactivated or destroyed [86]. Genes annotated as hypothetical/unknown are typically handled via a weight-of-evidence approach (e.g., domain and homology searches against curated databases and absence of known virulence/AMR markers) and may trigger additional review rather than automatic acceptance or rejection. Phage manufacturing avoids lysogenic strains and uses sensitive technologies like PCR or shotgun sequencing to detect any bacterial DNA or non-functional genetic material from purified viral preparation. Potency and host range characterization are considered critical quality attributes. The phage’s potency is assessed by its infectious titer (usually quantified in plaque-forming units, PFU) under established assay conditions [88]. Every batch is routinely tested for active phage concentration using double-layer agar plaque assays and spot testing on a reference strain. To ensure potency remains within specifications during the intended shelf life, stability-indicating assays should be established in accordance with the International Council for Harmonisation of Technical Requirements for Pharmaceuticals for Human Use (ICH) recommendations [89]. At least four ICH stability characteristics are frequently checked (appearance, infectious titer, sterility/microbiological purity, and pH) under established storage settings [90]. Manufacturers must test each phage against a panel of bacterial strains, including isolates of the target pathogen and allied species, to determine its lytic activity spectrum. These tests [91] (usually by spot or plaque assays on agar) ensure that the phage infects the desired targets and help establish the product label and risk assessment (e.g., absence of off-target effects). When testing phages for specific markets, it is important to incorporate local pathogen strains [92].

In-process and release controls are crucial for both active ingredients and finished products. Numeric acceptance criteria for purity and potency are generally product-specific and regulator-dependent (e.g., defined PFU specifications under validated assay conditions; predefined limits for residual host-derived impurities such as host DNA and endotoxin where relevant) and may not be fully publicly disclosed for commercial preparations. The minimum expectation is that these specifications are pre-defined, validated, and supported by stability-indicating data, while matrix-validated performance is demonstrated under intended conditions of use (matrix, dose/titer, contact time, and temperature). The active material (purified phage bulk) is tested for identification (genomic fingerprint or sequencing) and sterility/microbial purity to guarantee the absence of any adventitious bacteria or endotoxins. Analytical tests for phage purity typically check for the absence of bacterial DNA or endotoxin (particularly critical when using lytic products of Gram-negative cultures) and establish that only the desired phages are present. The EMA draft guideline recommends conducting potency tests at several phases (seed lot, bulk, final product) to ensure uniformity in titer and infectivity. These assays are calibrated using reference standards (phage and host master banks). Good Manufacturing Practice (GMP) standards apply to biologics, including process validation studies to determine essential parameters and hold times. Each lot must meet release criteria for sterility, identification, and titer before use. For several commercially available products, detailed batch release specifications and acceptance limits are not fully available in the public domain; therefore, this review focuses on the quality attributes that are consistently expected to be defined and auditable.

From a practical food bioprocess perspective, formulation and delivery strongly influence observed performance: the stability of infective titers can be affected by matrix physicochemical conditions (e.g., pH, ionic strength, organic load), temperature during distribution, and contact time on the target surface [3,93]. Consequently, ‘better efficacy’ in real-world applications usually reflects improved maintenance of infective titer and contact with target cells under the intended use conditions (e.g., optimized carriers/buffers, protective excipients, or delivery formats that enhance persistence on surfaces), rather than intrinsic differences in lytic capacity alone.

Overall, the production and characterization of phage products combine traditional virology with current molecular quality control. Effective seed stock management, host culture control, contaminant elimination, and genetic uniformity are critical. If the finished product is a cocktail, makers must show that each component’s identity and activity are preserved in the mixture. The Organisation for Economic Co-operation and Development (OECD) and EMA guidance materials underline the importance of thoroughly documenting the biological activity and stability of each phage. Phage quality parameters like identity (genome and phenotype), efficacy (infectious titer), purity (microbial and genetic), stability, and defined host range must all be monitored with the same rigor as conventional biological treatments.

## 6. Global Regulatory Landscape and Data Standards

Regulatory frameworks for phage-based food biocontrols vary substantially across jurisdictions. While core quality and safety principles (e.g., identity, purity, potency, genomic suitability, and consistency) are broadly similar, regulatory classification and data requirements are not identical across animal/food-related versus plant/agricultural uses. In many jurisdictions, plant applications are assessed under biopesticide/plant protection frameworks, whereas food and animal-related uses may fall under food law, feed additive, or veterinary/animal health pathways depending on intended use [2,8].

In the United States, most food-use phages are evaluated by the FDA primarily through the GRAS pathway for processing aids, while the USDA/FSIS provides additional oversight for applications in meat and poultry systems; notably, labeling is generally not required under these routes [94]. Depending on the intended use and mode of contact with food, U.S. authorization may proceed via distinct mechanisms, including GRAS notifications, Food Contact Notifications/Food Contact Substance routes for contact materials, or Food Additive Petitions for additives (Table 1). Beyond the U.S., regulatory pathways and/or reported official approvals have been described in multiple jurisdictions, including Switzerland, Israel, Canada, Australia, New Zealand, and China, while in other markets (e.g., Brazil and parts of Asia) the regulatory approach is more heterogeneous and frequently aligned with case-by-case or feed/zootechnical frameworks [65,95,96]. To synthesize these jurisdictional differences into an operational view, Table 2 summarizes the main regulatory touchpoints and oversight responsibilities across the phage product lifecycle.

Canada and Australia used a middle ground approach. The Food Directorate of Health Canada offers Letters of No Objection (LONO) for phage products after reviewing dossiers. In 2025, Cytophage Technologies gained LONO clearance for two products (OvaPhage™ for eggs and PhageFend™ for poultry), recognizing phage mixtures as safe and effective for food processing [97]. Thus, Canada basically classifies phages as novel food additives that require ministerial approval, in line with US precedence. Australia/New Zealand (via FSANZ) allows for the use of phages as “processing aids” [98]. FSANZ assessments (e.g., for *Salmonella*-targeting phages or *Listeria* phage P100) found that phages serve a technological role throughout processing and have established standards for identity and purity in Schedule 3 of the Food Standards Code [99]. FSANZ states that there are presently no phage standards in Codex Alimentarius, and thus use is determined by domestic risk assessment and consultation.

**Figure 2:**
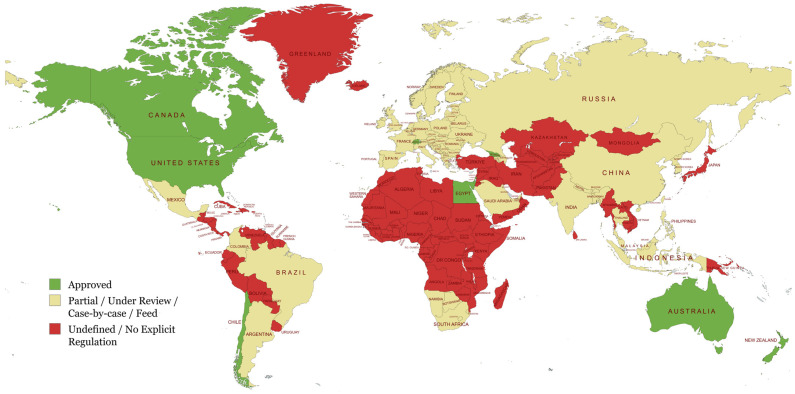
Global regulatory landscape for bacteriophage-based biocontrol in food systems (map created with MapChart.net, accessed on 13 March 2026).

In contrast, the European Union currently lacks dedicated phage regulation. Phage reviews by the EFSA and the EU Commission have been conducted on a case-by-case basis. In 2012, an EFSA BIOHAZ panel decided that Listex™ P100 is safe and effective against *Listeria*. However, legislation is still pending [100]. National authorities preferred to classify phages as “processing aids” (used just during manufacturing and serving no ongoing purpose in the final product) rather than food additives [101]. Overall, EU regulatory approval is cautious and gradual. The UK has taken a similar case-by-case approach (treating phages as processing aids under the Food Standards Agency’s jurisdiction) [102]. In Japan, the regulatory landscape is still growing but largely progressive. Government-funded research, such as the “Food Safety by Phage” initiative, has shown that phages are effective and safe in food [103]. Similarly, certain Latin American countries are starting to investigate phage biocontrol [104]. For instance, the INSPEKTOR product line, developed in Chile and designed to inhibit *Salmonella*, is already available in some Latin America countries. More broadly, several bacteriophage products are already marketed, particularly as veterinary/zootechnical solutions. However, regulatory oversight is typically implemented through existing national frameworks (e.g., feed additives, veterinary products, or biopesticides) rather than through harmonized, phage-specific food legislation. Consequently, authorizations are country-specific and heterogeneous, and publicly accessible regulatory identifiers are not consistently available. In practice, big markets frequently await U.S./EU precedent, and thus regulatory uncertainty in Latin America persists. Overall, as shown in Figure 2, the most clearly documented approvals/official acceptances to date are concentrated in North America, Australasia, and a few Asian nations, with the EU and many developing regions still establishing their frameworks. In the EU, classification and acceptance may differ by Member State and application context; the map reflects publicly documented positions/authorizations rather than a harmonized EU-wide authorization route.

Regardless of location, regulatory agencies demand comprehensive dossiers that validate phage identification, quality, and safety. As detailed in Section 5, the technical data required to support these applications, specifically genomic integrity and potency, are increasingly standardized across jurisdictions. However, while technical requirements are converging toward shared standards (e.g., use of whole-genome sequencing (WGS) and standardized PFU assays), legal classifications remain fragmented. The same phage preparation, characterized by identical stability and purity data, may be categorized as a processing aid, a food additive, or a biopesticide depending on the target market [91]. This regulatory divergence creates significant market access uncertainty, as manufacturers must tailor their submissions to fit disparate legal definitions despite using consistent quality control metrics.

Harmonizing phage rules around the globe is difficult. Phages fall into several legal categories: they can be considered food additives (if active in food), processing aids (if active during manufacture), feed or seed additives, or even biopesticides (if used on crops or animals). Phages are allocated to different channels in different nations (for example, the US FDA/USDA, the EPA, and the Canadian Food Inspection Agency (CFIA)). Listex™ is GRAS in the US but only has informal clearance in the EU due to processing-aid interpretation. Furthermore, governments mandate different data formats and acceptability standards. The EU has long EFSA reviews, while the US, Canada, and Australia have more efficient processes. Differences in legal terminology (e.g., “ongoing technological function” vs. “additive effect”) might alter a product’s classification (FSANZ’s analysis is an example). Infrastructure constraints also provide challenges: many areas lack certified phage manufacturing facilities and specialist testing labs. Intellectual property and labeling difficulties (for example, whether phages are “genetically modified” or “organic”) hinder approvals. In effect, non-standardized rules and procedural lag (particularly in the EU) have slowed the global adoption of phage biocontrols.

In practice, non-standardized standards and procedural lag (especially in the EU) have hindered the global use of phage biocontrols. It is also worth noting that some regions, such as former Soviet republics (e.g., Georgia and Ukraine), have a long history of phage use, notably in human medicine [105,106]. However, while these countries have substantial expertise, their formal legal classifications for phage usage in food systems are frequently ambiguous or classified outside of typical Western regulatory frameworks, adding to the geographic difference shown in Figure 2.

Major international organizations are starting to address phage regulation, but loopholes remain. Codex Alimentarius presently does not have a defined standard for phage biocontrols, either in its General Standard for Food Additives or in processing aid guidelines. The World Health Organization (WHO) (with its AMR One Health programs) and FAO advocate alternatives to antibiotics, and phages are mentioned in AMR discussions, although there is no specific Codex code of practice for phages in food [31]. The OECD has created a Guidance Document on bacteriophage use in plant protection, which shows how phage active ingredients can be evaluated using existing pesticide frameworks (including identity, efficacy, and environmental destiny studies) [107]. In Europe, the EMA has produced draft recommendations for phage therapy products (medical applications) [86], while the European Pharmacopoeia Commission has adopted general chapters on phage therapy (indicating greater acceptability) [108]. These activities demonstrate rising institutional support for harmonized standards. Nonetheless, a truly worldwide “One Health” oversight mechanism for phage biocontrols has not yet been devised. To summarize, present international entities promote uniform data (genome sequences, purity assays, efficacy testing), although coordination is still developing.

## 7. Ethical and Societal Governance for Live Biocontrol

The deliberate release of bacteriophages as self-replicating biocontrol agents involves distinct ethical problems. Unlike chemical sanitizers, phages can evolve, spread, and even transfer genetic material in the environment, making the results unpredictable. Key concerns include the difficulties of limiting or reversing ecological releases, the unknown effects on microbial communities, and whether full informed permission can ever be acquired when phages are used at the ecosystem or population level. Experts suggest that oversight of living interventions should take into account their unique and dynamic nature [109]. Governance frameworks emphasize the need for tailored risk assessment and containment mechanisms proportionate to the intended use of live biocontrol agents (and, where relevant, engineered variants) in food systems [110]. In reality, this means that any phage biocontrol program must include rigorous safety testing, phased trials, and explicit responsibility (for example, determining who is liable if phage dissemination causes unintentional injury). In food industry settings, these ethical concerns translate into concrete obligations for manufacturers and operators: defined conditions of use, traceable quality systems, and clear accountability for monitoring, corrective actions, and communication.

Self-amplification and mutation may cause phages to behave differently over time, confounding risk–benefit assessments in a context-dependent manner. In food chain applications, the practical ethical relevance is therefore the need for proportionate lifecycle monitoring and predefined corrective-action triggers when performance drifts (e.g., reduced efficacy or resistance signals), particularly where broader environmental release is plausible (e.g., via facility wastewater). In many cases, consumers and communities are unable to avoid encountering biocontrol agents on food or in the environment, creating concerns regarding individual and communal decision-making. Once unleashed, phages may be tough to eliminate. Ethical governance necessitates long-term monitoring, public reporting of outcomes, and preparations for problem resolution if they develop.

Public adoption of phage biocontrol is dependent on trust and clear disclosure. Many customers are unfamiliar with phages and may link the term “virus” with disease, causing intuitive uneasiness. According to survey data, some customers are concerned about the safety of live viruses in food, while others may be prepared to pay for phage-treated fruit [81]. Education can influence attitudes, as seen by an increase in comfort with bacteriophage additions [111]. To foster confidence, stakeholders prioritize transparency. In several jurisdictions, where phage preparations are classified as processing aids, on-pack labeling is generally not required; nevertheless, transparent public information and non-misleading communication remain important to maintain trust. Approaches to disclosure vary, and stakeholders continue to debate the most appropriate mechanisms for informed choice and public reassurance [81,112]. Experts point out that low awareness and literacy about phage science is a major barrier to acceptance. To address this, public outreach, clear messaging about phage safety (e.g., phages do not infect human cells), and dialog platforms to address concerns are necessary.

In food industry practice, ethical governance for phage biocontrol is operationalized through (i) evidence-based and non-misleading claims aligned with dossier-tested conditions of use, (ii) stewardship to prevent use as a substitute for baseline hygiene/HACCP controls, (iii) clear accountability and triggers for action when performance drifts (e.g., reduced efficacy or resistance signals), (iv) transparency to stakeholders even where “processing aid” classification limits on-pack labeling, and (v) proportional environmental responsibility for plausible release routes (e.g., facility wastewater streams).

A variety of governance approaches can aid in the ethical application of phage biocontrol. Robust review, clear regulations, and broad oversight are all critical factors. Proposals for food-use phage biocontrol should be evaluated by the competent regulatory authorities and, where applicable, appropriate oversight mechanisms consistent with the jurisdiction and use scenario. This includes extensive risk–benefit analyses, long-term monitoring strategies, and publication of safety data prior to and following approval.

Phage biocontrol is frequently promoted within a One Health paradigm, which recognizes connections between human, animal, and environmental health [11]. A One Health strategy advocates the use of phages to minimize pathogen burdens throughout the food chain and on farms. In principle, this comprehensive vision might result in significant public benefits by reducing antibiotic resistance and increasing global food safety. However, the use of phage technologies must be evaluated against biojustice considerations, which include ensuring that benefits and burdens are spread fairly [113]. For example, proponents believe that phages have equity-promoting properties: they are low-cost, do not require an expensive cold chain, and may frequently be isolated locally [114]. Phages, with their inexpensive production costs and ease of use, can be spread more fairly than other emerging biomedical therapies. This shows that phages may, in time, increase access to safe food and medicine in resource-constrained environments.

Simultaneously, attention is required to avoid repeating biocolonial or exclusionary practices. If phage resources (such as environmental strains) are mostly collected in biodiverse impoverished countries, international governance must assure equitable benefit-sharing, similar to the objectives of the Nagoya Protocol [115,116]. Ethical standards for microbiome research underline the importance of involving indigenous and local populations in decision-making and allowing participants to share in any benefits. By analogy, phage biocontrol projects should involve local stakeholders in co-managing bioprospecting to guarantee that small farmers or indigenous communities benefit from new technology rather than being marginalized. In brief, the justice factor necessitates that One Health objectives do not disregard equality. Policies must be specifically geared to protect disadvantaged groups, increase access (for example, through public health campaigns in low-income nations), and prohibit exploitation of biological resources.

Regulatory strategies for live biocontrol should draw on well-established policy ideas. Many argue that phage usage should be careful due to uncertainty about ecological and evolutionary implications [117]. This includes rigorous pre-approval review and low risk exposure until safety is shown. The subsidiarity principle (which is significant in international One Health strategy) proposes that decisions be made at the most effective governance level. The EU’s zoonoses regulation balances EU-wide norms with national actions [118], while phage deployment may need localized decision-making (e.g., state or provincial control of field trials) within a harmonized worldwide framework. Proportionality is also important. Regulatory requirements (such as clinical studies, environmental evaluations, and labeling guidelines) should be proportionate to the amount of risk and scope of application. Trivial or highly limited uses (such as surface treatment of packaged goods) may require less control than large-scale environmental emissions. Finally, legitimacy requires policies to be evidence-based, transparent, and inclusive. Phage governance is more credible and fair when data are openly disclosed, choices are justified, and affected publics are engaged. Adhering to these principles can help navigate the complexities of biocontrol deployment, such as requiring stepwise trials under the precautionary principle, coordinating policies across jurisdictions (subsidiarity), and avoiding overly burdensome rules that may stifle innovation (proportionality).

Phage biocontrol also exposes trade-offs between innovation and regulation, particularly in the areas of intellectual property and open science [119]. Most jurisdictions do not allow patents on naturally occurring species, including wild phages and their genomes. This means that firms cannot patent naturally occurring phages. Patents can be obtained for engineered phages, changed genomes, and specialized phage cocktails. This creates a paradox: on the one hand, broad access to natural phages is legally available [120], which may encourage open sharing and egalitarian use; on the other hand, innovation may lean toward complicated proprietary goods (akin to genetically altered crops) in order to secure investment returns. Some suggest that phages should be recognized as public goods [121]. Government or non-profit phage libraries can be sponsored to ensure a diverse range of strains are freely available. Public support for phage collections and shared databases has been proposed to address market reluctance to invest when exclusive rights are constrained. It is difficult to strike the right balance between open access and development incentives. Private companies argue that without exclusivity or compensation, they cannot justify the cost of registering or enhancing their goods. Alternatives have been proposed, including “pull” incentives such as government rewards or transferable patent extensions to stimulate R&D in socially useful phage uses.

Meanwhile, open-science supporters argue for maximum data sharing: sequencing phage genomes and publishing techniques in public repositories can hasten discovery while reducing duplication of effort. Any policy mix must consider the concerns of all stakeholders. For example, a system of standardized, publicly curated phage libraries (with agreed-upon quality criteria) could serve both open-access and commercial purposes, as long as contributors and source communities are properly recognized. The discussion concerning innovation policy continues to believe that phage biocontrol would thrive under a “commons” paradigm with shared stewardship, while others say that limited IP rights are required to activate the biotechnology industry. In practice, mixed models are likely, requiring careful governance to guarantee that the benefits of phage innovation (safer food, lower antibiotics) are not undercut by conflicts over patents or access.

## 8. Recommendations for Authorities and Industry

Global implementation of phage-based food biocontrol will require convergent policies and collaborative frameworks that extend beyond current regulatory silos. Although several phage products have reached food safety markets, regulatory scope, evidentiary expectations, and monitoring practices remain uneven across jurisdictions. Given jurisdictional differences in risk appetite, the objective is not identical decisions on specific phage products, but improved comparability through convergence on a minimum evidence package and interoperable dossier cores. A One Health framework can support food chain deployment by linking interventions to human, animal, and environmental protection goals [11]. The recommendations below are therefore organized along the full product lifecycle and translate harmonization objectives into concrete responsibilities for both authorities and manufacturers. Table 3 provides the operational map for this lifecycle governance, spanning characterization, pre-market evaluation, authorization, post-market monitoring, change control, and communication.

In this context, “minimum standards” refer to a core evidence package: WGS-based identity/suitability (excluding lysogeny and undesirable genes), defined potency/titer specifications with stability-indicating monitoring, purity controls (including residual host material and endotoxin where relevant), matrix-relevant performance verification, and bridging requirements for like-for-like cocktail updates.

Regulatory agencies should take the lead in developing a comprehensive international framework for phage biocontrol oversight. First, they should create common phage-specific standards and definitions: Agencies from different locations must agree on key nomenclature (e.g., what defines a phage active ingredient or a biocontrol “cocktail”) and safety and efficacy standards. However, requirements and review stringency vary widely. Harmonized dossier forms and data standards would allow a phage product that meets the criterion in one jurisdiction to be more easily assessed elsewhere. A shared common application dossier format for phage-based food safety products (similar to ICH common technical documents in pharmaceuticals) should be implemented to cover quality, safety, and claim substantiation in a consistent manner.

Second, they should set up interoperable phage seed banks [122] and master-file schemes: Regulatory agencies should work together to establish a worldwide network of quality-certified phage repositories. To avoid monopoly or undue IP constraints, such infrastructures can be implemented as federated, multi-center repositories and/or confidential master-file mechanisms that support traceability and comparability while preserving competition and proprietary development. New guidelines now demand the use of a traceable phage master bank [114]. By certifying phage seed batches through authorized reference centers (perhaps under WHO or OIE coordination), regulators can allow manufacturers to select from a pool of licensed phages with confidence in their safety and provenance. This paves the way for “dynamic phage bank” licensing, which allows a corporation to add a pre-certified phage to an existing cocktail without redoing the full certification procedure. Pilots in the United States and Europe have hinted at this flexibility, and formal approval of such bridging regulations for cocktail reformulation is critical.

Given that phages affect food safety [16,26,75,102], veterinary medicine [123], and the environment [124], regulatory organizations should establish collaborative forums or task groups to ensure that oversight is coherent across sectors where relevant, while allowing for sector- and jurisdiction-specific implementation. For example, an international organization may form a specialized Environmental and Food Phage Applications Working Group to draft unified advice (in addition to existing work for human medicinal phages). Ideally, phage biocontrol rules would be unified by organizations such as the Codex Alimentarius [125] or OECD [107], integrating phage use into global microbial risk management plans.

National and regional food safety authorities, including risk assessors and inspectors, are responsible for putting legislation into action on the ground. They should create and enforce practical criteria to ensure that phage biocontrol is safely and consistently implemented in food production. First, they should provide unambiguous criteria for efficacy claims and usage parameters: Authorities must clarify the claims of phage-based products (e.g., “reduces *Listeria* by 3 log CFU on ready-to-eat meats”) and the proof required to back up those claims. Standardizing these concepts across jurisdictions would reduce uncertainty and increase confidence. For example, whether phages are classified as “processing aids,” “food additives,” or biological control agents should be explained and standardized internationally [7,102]. Consistent criteria for efficacy (such as minimal log-reduction under defined settings) and safety (such as the requirement for the absence of transferable resistance genes) should be disclosed, preferably as joint guidance from major agencies. Harmonization in this sector ensures that food firms in different nations confront identical standards, and that phage-treated foods are allowed in international trade.

Food safety organizations should use phage-specific risk assessment frameworks [126], acknowledging that zero-risk cannot be achieved but may be managed scientifically. Building on recent risk profiling studies, authorities can create guidelines for evaluating phage applications (spraying, packing, surface cleaning, and so on) under realistic conditions. Furthermore, “phagovigilance” should be integrated into food safety oversight: companies that use phages in production may be required to report any loss of efficacy or unusual microbiological findings (for example, emergent phage-resistant strains or horizontal gene transfer events), allowing authorities to respond with updated guidance.

Because phages are living viruses added to foods, public perception can be an impediment if not addressed proactively. To increase openness, food safety authorities may consider consistent labeling or notification rules for phage-treated foods. Clear labeling of phage use can increase consumer trust and provide informed choices [112]. Regulatory bodies may, for example, allow for the use of a label statement such as “treated with bacteriophage to enhance safety,” supported by education campaigns demonstrating that phages are natural and safe to consume. In circumstances where phages are classified as processing aids (and hence not labeled), authorities should still inform the public about their use using websites or QR codes.

Phage producers and developers are at the forefront of bringing biocontrol products to market, and they play a critical role in establishing quality and consistency from the bottom up. First, they must adhere to high manufacturing and quality standards throughout the industry: Manufacturers should proactively implement GMP [127] or equivalent quality systems for phage production, especially in places where such standards are not yet required for food-use phages. Industry can facilitate regulatory clearances in many areas by ensuring all phage active ingredients meet internationally established quality norms. Companies are encouraged to deposit well-characterized phage strains in accredited repositories or work together to create a shared phage seed bank resource. An interoperable repository of validated phage reference strains for major foodborne pathogens can reduce duplication of isolation efforts and simplify regulatory review. Industry consortiums or public–private partnerships (perhaps funded by government funds) might lead to the development of such phage libraries [128,129,130] for common targets such as *Salmonella*, *Listeria*, and *E. coli*, with agreed-upon storage and data sharing protocols.

Phage firms should anticipate the paperwork requirements of various regulatory regimes and create complete common dossiers. Manufacturers facilitate regulatory convergence by exceeding minimum local criteria and aiming for a shared core dossier. Companies must also prepare for cocktail flexibility, which entails designing product lines such that individual phages can be swapped or introduced with minimal disturbance. Phage manufacturers should also participate in voluntary data sharing initiatives, such as contributing to an open database of phage genome sequences and phenotypic traits or sharing non-proprietary safety data, in order to create a collective evidence base that benefits the entire field and assuages regulators’ concerns [131].

Manufacturers, as producers of this technology, are responsible for monitoring and communicating the performance of phage biocontrol. They should set up internal phagovigilance programs to monitor how well their products operate once they are released to the market, gathering input from food processors on efficacy and any difficulties. Any evidence of decreased efficiency (such as a target bacterium developing resistance) should prompt studies and be disclosed to authorities so that solutions (e.g., changing the phage cocktail) can be implemented. Participating in international standardization efforts is also key: phage companies could help draft industry standards or codes of practice (under ISO or IAFP auspices) that codify best practices in phage production, handling, and application. Public health institutions can complement regulatory oversight by integrating phage-related signals into existing foodborne surveillance and AMR monitoring programs where relevant. Any such activities should be proportionate to the use scenario and rely on auditable reporting rather than continuous direct sampling.

## 9. Conclusions

The evidence synthesized in this narrative review indicates that bacteriophage-based biocontrol is technically feasible for reducing key foodborne pathogens across multiple food system contexts, including direct product applications and hotspots in processing environments. Its primary operational advantage is focused antibacterial action, which can be incorporated as an additional barrier within existing food safety processes. Nonetheless, sustained scale-up is now constrained by governance and standards rather than proof-of-concept efficacy alone. Across the literature and publicly documented regulatory expectations, a convergent technical baseline is repeatedly observed for acceptable food-use phage products: genome-resolved identity and confirmation of a strictly lytic profile, the absence (or defensible management) of genetic liabilities, potency defined by infective activity under specified assay conditions, matrix-relevant performance evidence, quantified purity with limits for residual host-derived impurities, and stability profiles that support labeling. The underlying challenge is that these components are not uniformly operationalized, which fragments authorization processes, complicates cross-border deployment, and undermines incentives for investing in high-quality systems. A One Health framework is presented here as a governance lens to support ongoing lifecycle stewardship and cross-sector coherence where relevant, rather than as a formal prerequisite for regulatory approval. Because biological performance varies with bacterial ecology and operational circumstances, proportionate post-market verification, susceptibility surveillance, and explicitly specified triggers for remedial actions are critical to maintaining efficacy and legitimacy. Improved cross-jurisdictional comparability may be best supported through interoperable dossier cores, defined comparison standards for cocktail adaptation, traceable seed bank infrastructures, and coordinated transparency approaches that maintain public trust without exaggerating claims. If lifecycle-oriented standards are implemented coherently by authorities and industry, phage biocontrol could become a routinely auditable, internationally deployable component of preventive food safety management and AMR mitigation.

## Figures and Tables

**Table 1:** Bacteriophage-based commercial products for food/feed biocontrol and their regulatory status.

Product Name	Target Pathogen(s)	Application/Food Matrix	Regulatory Status	References
**Listex™ P100**	*Listeria monocytogenes*	RTE meats, dairy, seafood	GRAS (GRN No. 198 & No. 218)	[40,46]
**PhageGuard S**	*Salmonella* spp.	Poultry, meat surfaces	Notified Processing Aid	[47]
**PhageGuard E**	*E. coli* O157:H7	Beef, cheese	Notified Processing Aid	[48]
**SalmoFresh™**	*Salmonella* spp.	Poultry, vegetables	GRAS (GRN No. 435)	[49]
**ListShield™**	*Listeria monocytogenes*	RTE meats	GRAS (GRN No. 528), 21 CFR §172.785	[50]
**EcoShield™**	*E. coli* O157:H7	Ground beef, leafy greens	FCN No. 1018, FSIS Directive 7120.1	[51]
**BAFASAL^®^**	*Salmonella* spp.	Animal feed	Notified Veterinary Biocontrol	[52]
**ShigaShield**	*Shigella* spp.	Food	GRAS (GRN No. 672)	[53]
**AgriPhage™**	*Xanthomonas* spp., *Pseudomonas syringae*	Plants, Production	US Environmental Protection Agency (EPA), EPA Reg. No. 67986-1 EPA Est. No. 67986-UT-001	[54]
**SalmoLyse^®^**	*Salmonella* spp.	Poultry (Live Animals/Feed)	GRAS (AGRN 74) status by the FDA CMV	[55]
**INSPEKTOR^®^**	*Salmonella* spp.	Poultry (Live Animals/Feed)	Notified Veterinary Bio-control	[56]
**FORMIDA^®^**	*E. coli*	Poultry (Live Animals/Feed)	Notified Veterinary Bio-control	[57]
**PHAGEIN^®^**	*E. coli and Salmonella* spp.	Calves (Bos taurus) (Live Animals/Feed)	Notified Veterinary Bio-control	[58]

**Table 2:** Proposed operational responsibilities for regulatory authorities and industry stakeholders across bacteriophage product lifecycle.

Domain	Core Data Expected Across Jurisdictions
**Genomic Integrity**	Full-coverage sequencing, confirmation of strictly lytic lifestyle, absence of integrase, lysogeny modules, virulence factors, toxin genes, and AMR determinants
**Identity & Potency**	Sequence-tied identity assays; infectivity-based potency (PFU), host range validation against relevant strains, efficiency-of-plating panels.
**Purity**	Quantification of residual host cell proteins and DNA, confirmation of absence of viable production host, excipient identity and acceptable limits.
**Stability**	Real-time and accelerated stability profiles under labeled storage and in-use conditions, potency retention models; delivery system compatibility
**Exposure Assessment**	Scenario-based estimates of infective titers at point of consumption, worker exposure evaluations; environmental fate and inactivation pathways

**Table 3:** Operational responsibilities for authorities and industry across the phage product lifecycle.

Stage	Authorities	Industry/Manufacturers
**Product Characterization**	Define minimum data standards (WGS-based identity/suitability; potency/titer specifications; purity controls incl. residual host DNA/endotoxin where relevant; stability-indicating assays)	Generate data packages using validated, matrix-relevant methods. Maintain traceable seed banks and batch records.
**Pre-Market Evaluation**	Review dossiers using protection goals and scenario-based exposure logic. Clarify acceptable uncertainty and required mitigations.	Provide complete dossiers with matrix-validated kill studies, stability models, exposure scenarios, and QC analytics.
**Authorization**	Specify scope of use, claims, and conditions (matrix, dose/titer at use, contact time, temperature). Define criteria and bridging data for ‘like-for-like’ cocktail updates.	Align labels with dossier-tested conditions. Ensure consistent manufacturing and batch-to-batch comparability.
**Post-Market Monitoring**	Require proportional early-phase sampling (residual titers, susceptibility trends, environmental checks). Trigger corrective actions when thresholds are exceeded.	Implement verification sampling, maintain potency and stability trend charts, conduct susceptibility surveillance, and update cocktails when needed.
**Change Control**	Provide guidance for bridging data needed for reformulation or new component substitution.	Prepare bridging packages (resequencing, host range comparisons, stability verification) and document all updates.
**Communication & Transparency**	Offer public guidance and coherent messaging on phage use and oversight logic.	Provide clear use instructions, stewardship commitments, and transparent performance summaries when requested.

## Data Availability

The original contributions presented in this study are included in the article. Further inquiries can be directed to the corresponding authors.

## Abbreviations

The following abbreviations are used in this manuscript:

AMR	Antimicrobial Resistance
ARGs	Antibiotic Resistance Genes
CFIA	Canadian Food Inspection Agency
CFU	Colony-Forming Unit
EFSA	European Food Safety Authority
EMA	European Medicines Agency
EPA	Environmental Protection Agency
FAO	Food and Agriculture Organization
FDA	Food and Drug Administration
FSANZ	Food Standards Australia New Zealand
GMOs	Genetically Modified Organisms
GMP	Good Manufacturing Practice
GRAS	Generally Recognized as Safe
IAFP	International Association for Food Protection
ICH	International Council for Harmonisation
ISO	International Organization for Standardization
LONO	Letter of No Objection
MOI	Multiplicity of Infection
OECD	Organisation for Economic Co-operation and Development
OIE	World Organisation for Animal Health (formerly Office International des Epizooties)
PFU	Plaque-Forming Unit
QC	Quality Control
RTE	Ready-to-Eat
UNEP	United Nations Environment Programme
USDA	United States Department of Agriculture
WGS	Whole-Genome Sequencing
